# Communication between Cyclin-dependent kinase Cdc2 and the Wis1-Spc1 MAPK pathway determines mitotic timing in *Schizosaccharomyces pombe*

**DOI:** 10.1242/bio.053322

**Published:** 2020-07-21

**Authors:** Agamani Ghosal, Priyanka Sarkar, Geetanjali Sundaram

**Affiliations:** Department of Biochemistry, University of Calcutta, 35, Ballygunge Circular Road, Kolkata, 700019, WB, India

**Keywords:** Cdc2, Spc1, *S. pombe*, Rad24, Wis1, Mitosis

## Abstract

Checkpoint activation and gene expression modulation represent key determinants of cellular survival in adverse conditions. The former is regulated by cyclin-dependent kinases (CDKs) while the latter can be controlled by mitogen-activated protein kinases (MAPKs). Association between cell-cycle progression and MAPK-dependent gene expression exists in cells growing in optimal environments. While MAPK-mediated regulation of the cell cycle is well characterised, the reciprocal influence of mitotic CDK on stress response is not well studied. We present evidence that CDK activity can regulate the extent of MAPK activation in *Schizosaccharomyces pombe* cells. We show that increasing or decreasing mitotic CDK (Cdc2) activity in *S. pombe* cells can affect the activation of stress responsive MAPK (Spc1) even in the absence of stress stimuli. Our results indicate that the strong correlation between Cdc2 activity and Spc1 MAPK-activity in *S. pombe* is important in regulating mitotic timing.

This article has an associated First Person interview with the first author of the paper.

## Abstract (precis)

**Summary:** Modulation of Cdc2 activity can change the extent of Spc1 activation and preserve mitotic fidelity in *Schizosaccharomyces pombe* cells.

## INTRODUCTION

During adverse conditions like oxidative stress, heat shock, DNA damage, osmotic imbalances and nutrient limitation, eukaryotic cells defend themselves by activating the expression of several genes involved in repair and prevention of the damage caused by such stimuli (Shiozaki and Russell, 1995; Samejima et al., 1997; Shiozaki et al., 2017; Vivancos et al., 2006, 2004; Toone et al., 1998; Day and Veal, 2010; Sundaram et al., 2008; Saitoh and Yanagida, 2014; Shiozaki and Russell, 1996; Shieh et al., 1998). Simultaneously, they also activate checkpoints that delay cell cycle progression, thereby providing the extra time essential for such repair activities (Shieh et al., 1998; Alao and Sunnerhagen, 2008; Yuan et al., 2018; Chaudhuri et al., 2005; Rothe et al., 2017). Cellular survival under adverse conditions is dependent on both gene expression modulation and checkpoint activation. It is therefore not surprising that these two mechanisms are interdependent on each other. In *Schizosaccharomyces pombe* the Wis1-Spc1 mitogen-activated protein kinase (MAPK) pathway is known to be a major regulator of stress-responsive gene expression programs (Nguyen et al., 2002). The MAPK Spc1 (p38 homolog) has also been shown to regulate mitotic progression (Petersen and Hagan, 2005; Lopez-Aviles et al., 2008; Fan et al., 2005; Paul et al., 2018). The association of Spc1 with mitosis exists in unperturbed cells growing under optimal conditions as well (Shiozaki and Russell, 1995; Sundaram et al., 2008; Hartmuth and Petersen, 2009; Petersen and Nurse, 2007). We had earlier reported a very striking feature of this link (Paul et al., 2018). We discovered that Spc1 can sense hyperactivation of the cyclin dependent kinase Cdc2 (Cdk1 homolog) in *S*. *pombe*. Cdc2 activity determines whether the *S. pombe* cells will initiate mitosis or not. Tight regulation of Cdc2 activity is therefore critical for preserving mitotic fidelity. Cdc2 activity is mainly regulated by the positive regulator Cdc25 phosphatase (Millar et al., 1991) and the negative regulator Wee1 kinase (Kellogg, 2003; Tang et al., 1993). It is quite evident that the balance of Wee1 and Cdc25 function is critical for maintaining optimal Cdc2 activity and perturbations to this balance will result in aberrant mitotic timing. Our previous work suggested that such imbalances can be counteracted by Spc1 (Paul et al., 2018). It activates backup mechanisms that partially rescue the cells from the deleterious effects of aberrant mitotic timing resulting from Cdc2 hyperactivation. We showed that a key effector of this rescue mechanism is the 14-3-3 homolog Rad24, which is an indirect negative regulator of Cdc2. Rad24 is known to inhibit Cdc2 by exporting Cdc25 out of the nucleus (Lopez-Aviles et al., 2005; Smith et al., 2002). We also reported that Cdc2 hyperactivation is associated with an Spc1-dependent increase in *rad24*^+^ expression. This mechanism was found to be active exclusively in cells where both Cdc2 and Spc1 activities were significantly high. Activation of Spc1 in cells with basal Cdc2 activity failed to activate this mechanism and in fact *rad24^+^* expression decreased in these cells exhibiting a completely opposite outcome at the level of gene expression regulation. This observation raised the possibility that Spc1 functions can be affected by changes in Cdc2 activity. In this report we show that perturbing the balance of positive and negative regulators of Cdc2 to make Cdc2 hyperactive leads to increased activation of Spc1. Our results show that mutants with increased or decreased Cdc2 activity show corresponding changes in basal Spc1 activity even in the absence of any stress stimuli. Thus, Cdc2 hyperactivity represents a new trigger for Spc1 activation and possibly this connection functions as a mechanism for controlling Cdc2 activity. Our results further indicate that Cdc2-dependent changes in Spc1 activity are mediated via Wis1 MAP Kinase Kinase (MAPKK), which is known to phosphorylate and activate Spc1 in response to stress stimuli. Previously, the activity of the Spc1 binding partner Srk1 has been shown to be regulated in a cell-cycle dependent manner (Lopez-Aviles et al., 2005). Our observations, however, establish a correlation between Cdc2 and Spc1 activity, thereby providing a mechanistic explanation for cell-cycle dependent changes in Spc1 functions. While characterising these mechanisms, we found that nucleotide depletion caused by treating cells with the ribonucleotide reductase inhibitor hydroxyurea (HU) can also trigger an increase in Spc1 activity in a Wis1-dependent manner. Our results add interesting dimensions to the known landscape for connections between stress response and cell-cycle progression in eukaryotic cells.

## RESULTS AND DISCUSSION

### Perturbations to the Wee1/Cdc25 balance resulting in Cdc2 hyperactivation can trigger Spc1 activation

**Fig. 1.**
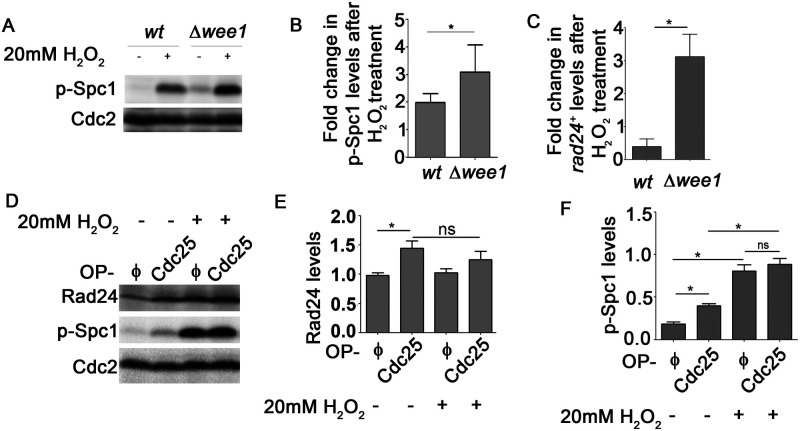
**Cdc2 hyperactivation results in increased Spc1 phosphorylation.** (A) Levels of Spc1 phosphorylation in wt and Δ*wee1* cells treated with 20 mM H_2_O_2_ for 15 min determined by immunoblotting and (B) quantified using ImageJ. *, *P*<0.05. Cdc2 levels were used for normalisation. (C) qPCR-based analysis of *rad24*^+^ expression in wt and Δ*wee1* cells treated with 20 mM H_2_O_2_ for 15 min. 18S rRNA expression was used for normalisation. *, *P*<0.05. (D) Rad24 and phosphorylated Spc1 levels in wt cells overexpressing either pGS008(φ) or pGS009 (Cdc25-GFP) after treatment with 20 mM H_2_O_2_ for 20 min. Quantification of both Rad24 (E) and phospho-Spc1 (F) was performed using ImageJ and Cdc2 levels were used for normalisation. *, *P*<0.05. All data are representative of three independent experiments. All bar graphs represent mean±s.e.m. A one-tailed *t*-test was performed for evaluation of statistical significance.

Earlier we had reported that Spc1 overexpression or increased Spc1 activity resulting from oxidative stress could increase *rad24^+^* expression exclusively in *Δwee1* cells that had a hyperactive Cdc2 (Paul et al., 2018). This increase in *rad24^+^* expression was not seen in wild-type (wt) cells (normal Cdc2 activity) under similar conditions. To investigate the reason for this observed difference between wt and *Δwee1* cells we looked for possible differences in the levels of Spc1 activity. We found that the levels of phosphorylated Spc1 (the active form of Spc1) were significantly higher in *Δwee1* cells. This difference existed not only when the cells were treated with 20 mM hydrogen peroxide (H_2_O_2_), but in untreated cells as well (Fig. 1A,B). This was very interesting (Spc1 levels under these conditions remained unchanged, see Fig. S1A). In accordance with our previous findings, we found that fold change in expression of *rad24^+^* mRNA after treatment with H_2_O_2_ was also higher in *Δwee1* cells (Fig. 1C). Cdc2 is known to be hyperactive in *Δwee1* cells. We decided to check whether the observed increase in Spc1 activity is associated with high Cdc2 activity by overexpressing Cdc25 in wt cells. We found that overexpression of Cdc25 to make Cdc2 hyperactive in wt cells also led to an increase in levels of active Spc1 (Spc1 levels under these conditions remained unchanged, see Fig. S1A). Rad24 levels in wt cells overexpressing Cdc25 were also higher (Fig. 1D–F). Our observations therefore clearly indicate that perturbing Wee1/Cdc25 balance to cause Cdc2 hyperactivation can trigger Spc1 activation in *S. pombe* cells even in the absence of stress stimuli. Interestingly, after H_2_O_2_ treatment there was no significant difference in phosphorylated Spc1 levels between wt cells transformed with the empty vector and wt cells overexpressing Cdc25 (Fig. 1D, lanes 3 and 4). Further, H_2_O_2_ treatment did not cause any change of Rad24 levels in cells overexpressing Cdc25 (Fig. 1D, lanes 2 and 4).

### Increased Spc1 activity in cells with perturbed Wee1/Cdc25 balance has a protective role

**Fig. 2.**
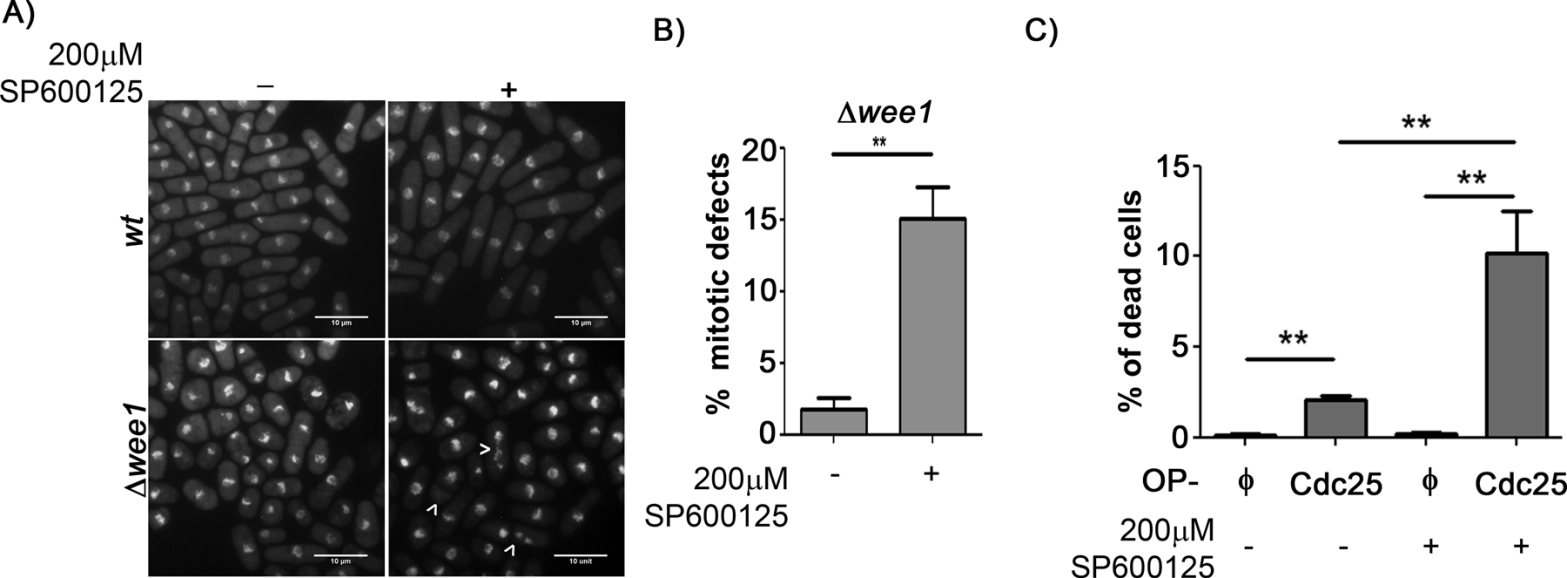
**Enhanced Spc1 activity protects cells from** **the** **deleterious effects of Cdc2 hyperactivation.** (A) Fluorescence images of wt and Δ*wee1* cells grown in the presence or absence of a pharmacological inhibitor of Spc1 (SP600125) for 2 h and stained with DAPI. Cells were stained with DAPI and processed for fluorescence microscopic imaging. Arrows represent defective chromosome segregation. Scale bars: 10 µm. (B) Quantification of abnormal mitotic events in Δ*wee1* cells following inhibitor treatment quantified using ImageJ. (C) Flow cytometric analysis of cell death (Propidium Iodide uptake) in wt cells overexpressing pGS008(φ) or pGS009(Cdc25-GFP) in the presence or absence of 200 µM SP600125 (Spc1 inhibitor) for 24 h. **, *P*<0.01. All data are representative of three independent experiments. All bar graphs represent mean±s.e.m. A one-tailed *t*-test was performed for evaluation of statistical significance.

We have reported earlier that increase in Spc1 activity partially rescues the *Δwee1* cells from premature mitotic entry (Paul et al., 2018). To further evaluate the significance of this mechanism, we treated *Δwee1* cells with a Spc1 inhibitor for 2 h (Hartmuth and Petersen, 2009). We found that inhibition of Spc1 leads to enhanced accumulation of mitotic defects in these mutants, clearly demonstrating the protective effects of increased Spc1 activity in *Δwee1* cells (Fig. 2A,B). Although the overexpression of Cdc25 was observed to increase Spc1 activity and Rad24 levels, these cells still entered mitosis prematurely. We therefore wanted to investigate whether increased Spc1 activation in these cells was having any protective effect at all. So we checked the viability of the cells overexpressing Cdc25 for 24 h, both in the presence and absence of the Spc1 inhibitor by measuring uptake of Propidium Iodide. We found that inhibition of Spc1 did not affect the viability of wt cells but caused a significant increase in cell death in cells overexpressing Cdc25 (Fig. 2C). This indicates that these cells require Spc1 to survive. Thus, although increased Spc1 activity could not rescue Cdc25 overexpressing cells from premature mitotic entry, it did provide some survival advantage, which, we found, is lost upon Spc1 inhibition.

### Cdc2 activity in unstressed *S. pombe* cells determines the basal activity levels of Spc1

**Fig. 3.**
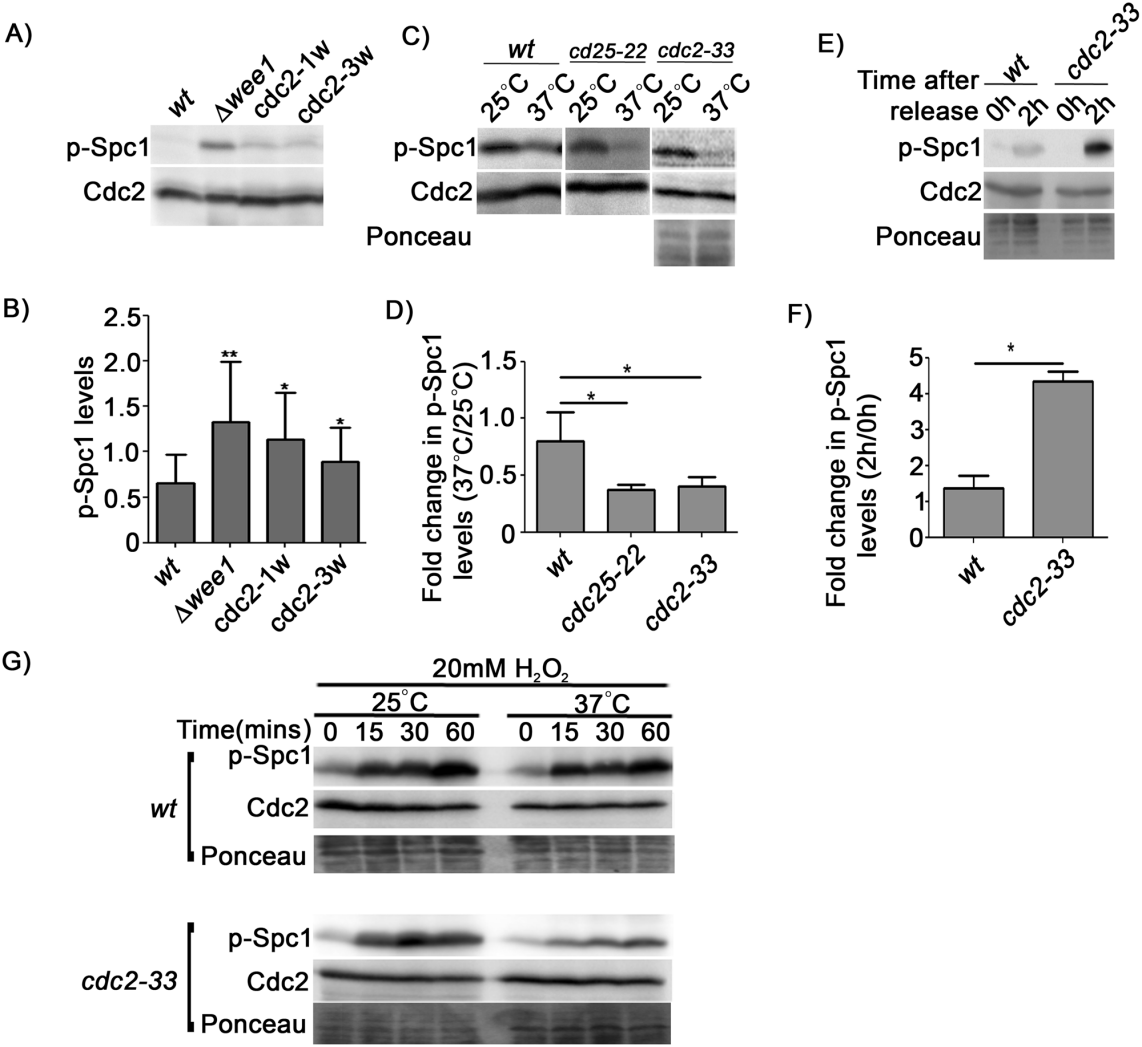
**Correlation between Cdc2 activity and levels of phosphorylated Spc1.** (A) Levels of phosphorylated-Spc1 in wt, Δ*wee1*, *cdc2-1w* and *cdc2-3w* cells determined by immunoblotting and (B) quantified using ImageJ, *, *P*<0.05; **, *P*<0.01. Cdc2 levels were used for normalisation. (C) Levels of phosphorylated-Spc1 in wt and *cdc25-22*, *cdc2-33 ts* mutants at the permissive (25°C) and non-permissive (37°C) temperatures determined by immunoblotting and (D) quantified using ImageJ. Cdc2 levels were used for normalisation in the case of wt and *cdc25-22*, and total protein in the case of *cdc2-33*, *, *P*<0.05. (E) Levels of phosphorylated-Spc1 in wt and *cdc2-33 ts* mutants before (0 h) and after release (2 h) into the permissive temperature after being arrested at the non-permissive temperature for 4 h, determined by immunoblotting and quantified using ImageJ. (F) Fold change in levels of phosphorylated Spc1 after release into permissive temperature for 2 h, * indicates *P*<0.05. Total protein levels were used for normalisation. (G) Levels of phosphorylated-Spc1 in wt and *cdc2-33 ts* mutants growing at either 25°C or arrested for 4 h at 37°C and treated with 20 mM H_2_O_2_ for the indicated time points determined by immunoblotting. Total protein was used as the loading control. All data are representative of three independent experiments. All bar graphs represent mean±s.e.m. A one-tailed *t*-test was performed for evaluation of statistical significance.

To confirm that high Cdc2 activity is a trigger for the observed increase in Spc1 activity, we checked the levels of phosphorylated Spc1 in various Cdc2 gain-of-function mutants (i.e. where Cdc2 is hyperactive), namely *cdc2-1w* (where the *cdc2* allele is insensitive to Wee1) and *cdc2-3w* (where the *cdc2* allele cannot be regulated by Cdc25) (Basi and Enoch, 1996; Caspari and Hilditch, 2015). We found phosphorylated Spc1 levels in all of these mutants to be higher than that of wt cells (Fig. 3A,B), (Spc1 levels under these conditions remained unchanged, see Fig. S1B). We repeated these experiments in *cdc25-22* and *cdc2-33 ts* mutants*.* These mutants harbour temperature-sensitive alleles of *cdc25^+^* and *cdc2^+^*, respectively. Consequently, at the non-permissive temperature, Cdc2 activity is low in both of these mutants. We found that shifting these mutants to the non-permissive temperature resulted in a decrease in levels of phosphorylated Spc1 (Fig. 3C,D), (Spc1 levels under these conditions remained unchanged, see Fig. S1B). It should be noted here that wt cells also exhibit a slight decrease in p-Spc1 levels, but the decrease is much more pronounced in both *cdc25-22* and *cdc2-33 ts* mutants. Thus, there seems to be a definite association between decreased Cdc2 activity and decreased Spc1 activity. We also found that this decrease in Spc1 activity was reversible, i.e. shifting the *cdc2-33 ts* mutants back to the permissive temperature resulted in increased levels of phosphorylated or active Spc1 (Fig. 3E,F), (Spc1 levels under these conditions remained unchanged, see Fig. S1F). Taken together, our results show that an increase or decrease in Cdc2 activity can cause a consequent increase or decrease of active Spc1 levels in the cells, thus clearly establishing an association between Cdc2 and Spc1 phosphorylation. To our knowledge, this is the first demonstration of such a connection. This kind of a cause-and-effect association between CDK and MAPK activity has not previously been reported.

Another remarkable observation is that complete inactivation of Cdc2 at a non-permissive temperature was shown to hinder Spc1 activation even after H_2_O_2_ treatment, unlike that in wt *S. pombe* cells (Fig. 3G). In fact chasing Spc1 activity for a period of 60 min following H_2_O_2_ treatment at the non-permissive temperature clearly indicated a suppression of Spc1 activation in *cdc2-33 ts* mutants. This further indicates a strong connection between Cdc2 activity and Spc1 function, even during stress response (Spc1 levels under these conditions remained unchanged, see Fig. S1G).

### Cdc2-dependent activation of Spc1 regulates the Rad24 concentrations of *S. pombe* cells

**Fig. 4.**
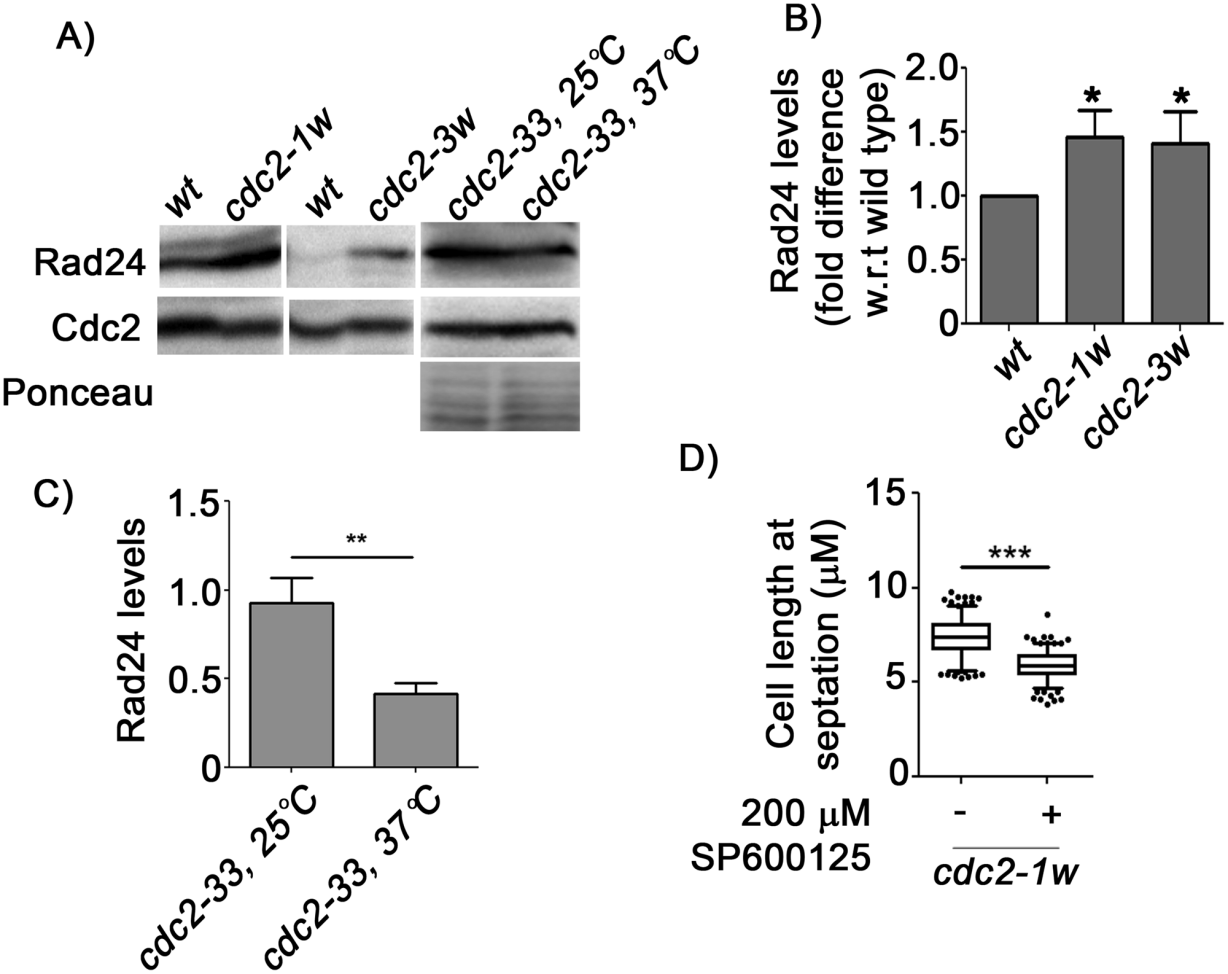
**Changes in Spc1 phosphorylation affect Rad24 expression.** (A) Rad24 levels in wt*, cdc2-1w*, *cdc2-3w* and *cdc2-33 ts* mutants (at 25°C and 37°C) determined by immunoblotting and quantified using ImageJ. (B) Fold change in Rad24 levels in *cdc2-1w* and *cdc2-3w* mutants with respect to that in wt cells, *, *P*<0.05. Cdc2 levels were used for normalisation. (C) Quantification of Rad24 levels in *cdc2-33 ts* mutants (at 25°C and 37°C), *, *P*<0.05. Total protein was used for normalisation. (D) Quantification of the length at septation of *cdc2-1w* mutant before and after treatment with Spc1 inhibitor (SP600125) for 2 h, using ImageJ software. ***, *P*<0.001 (*n*>200). All data are representative of three independent experiments. All bar graphs represent mean±s.e.m. A one-tailed *t*-test was performed for evaluation of statistical significance.

As shown in Fig. 3, variations in Cdc2 activity in different *cdc2* mutants lead to corresponding variations in the levels of active phosphorylated Spc1. We then investigated whether such differences affect the levels of Rad24 in these cells. We found that the Rad24 levels in these mutant cells also changed in direct correlation with the Spc1 activity. In *cdc2-33* mutants at the non-permissive temperature, the Rad24 levels were found to be low (Fig. 4A,C). In cells with hyperactive Cdc2, such as *cdc2-1w* and *cdc2-3w*, Rad24 levels were found to be high (Fig. 4A,B). We then treated the *cdc2-1w* mutants with a known pharmacological inhibitor of Spc1 for 2 h to validate the functional connection between Cdc2–Spc1 and Rad24 (Fig. 4D). In the *cdc2-1w* mutant, the *cdc2* allele is insensitive to Wee1 and therefore hyperactive. However, changing Cdc25 levels in this background can still affect Cdc2 function. Therefore, we would also expect Rad24-dependent Cdc2 regulation to be functional in this mutant, i.e. the Spc1-dependent mitotic regulation mechanism would be functional. We inhibited Spc1 in this mutant and found that mitosis was accelerated, thereby clearly indicating that Spc1 activity is essential for combating replication stress in these mutants.

### Wis1 transduces the perturbations in Cdc2 activity to Spc1

**Fig. 5.**
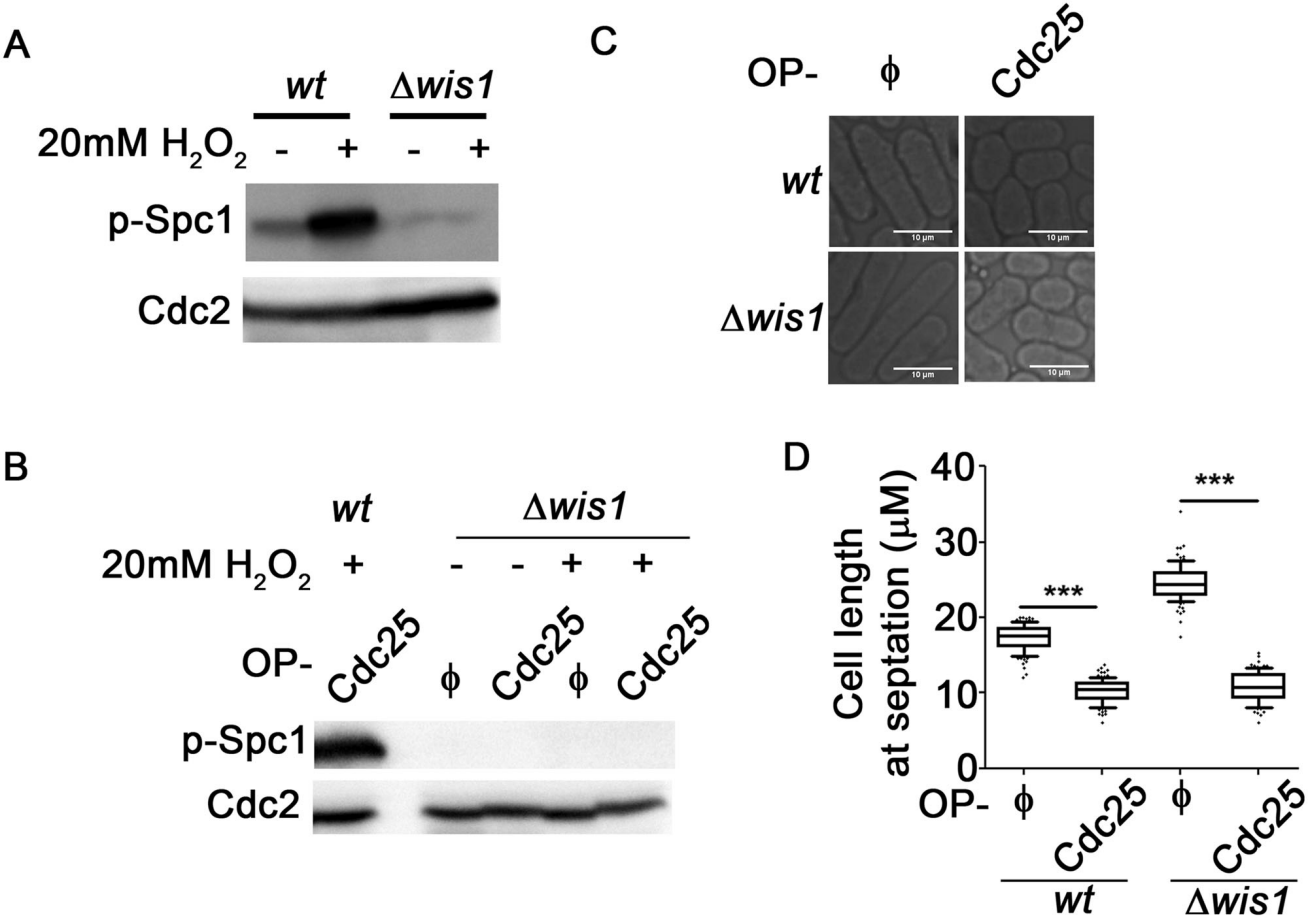
**Wis1 is essential for the connection between Cdc2 hyperactivation and Spc1 phosphorylation.** (A) Levels of Spc1 phosphorylation in wt and Δ*wis1* cells treated with 20 mM H_2_O_2_ for 15 min determined by immunoblotting. Cdc2 levels were used as loading control. (B) Levels of Spc1 phosphorylation in Δ*wis1* cells overexpressing pGS008(φ) or pGS009(Cdc25-GFP) after treatment with 20 mM H_2_O_2_ for 20 min, determined by immunoblotting. Lysates from wt cells overexpressing Cdc25 were loaded in parallel as a positive control of p-Spc1 signal. (C) Brightfield images of wt and Δ*wis1* cells overexpressing pGS008(φ) or pGS009(Cdc25-GFP). Scale bars: 10 µm. (D) Quantification of length at septation (*n*>200) of cells shown in C done using ImageJ software. ***, *P*<0.001. All data are representative of three independent experiments. A one-tailed *t*-test was performed for evaluation of statistical significance.

Activation of Spc1 by phosphorylation in *S. pombe* is brought about by the MAPK kinase Wis1. We therefore investigated whether Wis1 is a mediator for the connection between Cdc2 and Spc1 activities. Oxidative stress in *Δwis1* mutants does not cause an increase in phosphorylated Spc1 levels (Fig. 5A). Similarly, overexpression of Cdc25 in the absence of Wis1 is unable to mediate Spc1 activation (Fig. 5B), (Spc1 levels under these conditions remained unchanged, see Fig. S1C,D). Cdc2 hyperactivation via Cdc25 overexpression does occur in these cells, but they enter mitosis prematurely, dividing at shorter lengths (Fig. 5C,D). Notably, the extent of this shortening is greater in *Δwis1* than wt cells, clearly indicating that the inability to activate Spc1 in response to Cdc2 hyperactivation hinders the cell's ability to restrict the premature mitotic entry. Hence, we conclude that the Spc1 activation in response to misregulation in CDK function is Wis1 dependent. Wis1 has already been reported to be a substrate for the kinase Cdc2 (Swaffer et al., 2016). Possibly Wis1 activation via Cdc2 is essential for the observed Cdc2-dependent changes in Spc1 activity. However, further experiments with *Wis1* mutants that cannot be phosphorylated by Cdc2 are required to confirm that possibility.

### Replication stress caused by Cdc2 hyperactivation might be a reason for increased Spc1 activity

**Fig. 6.**
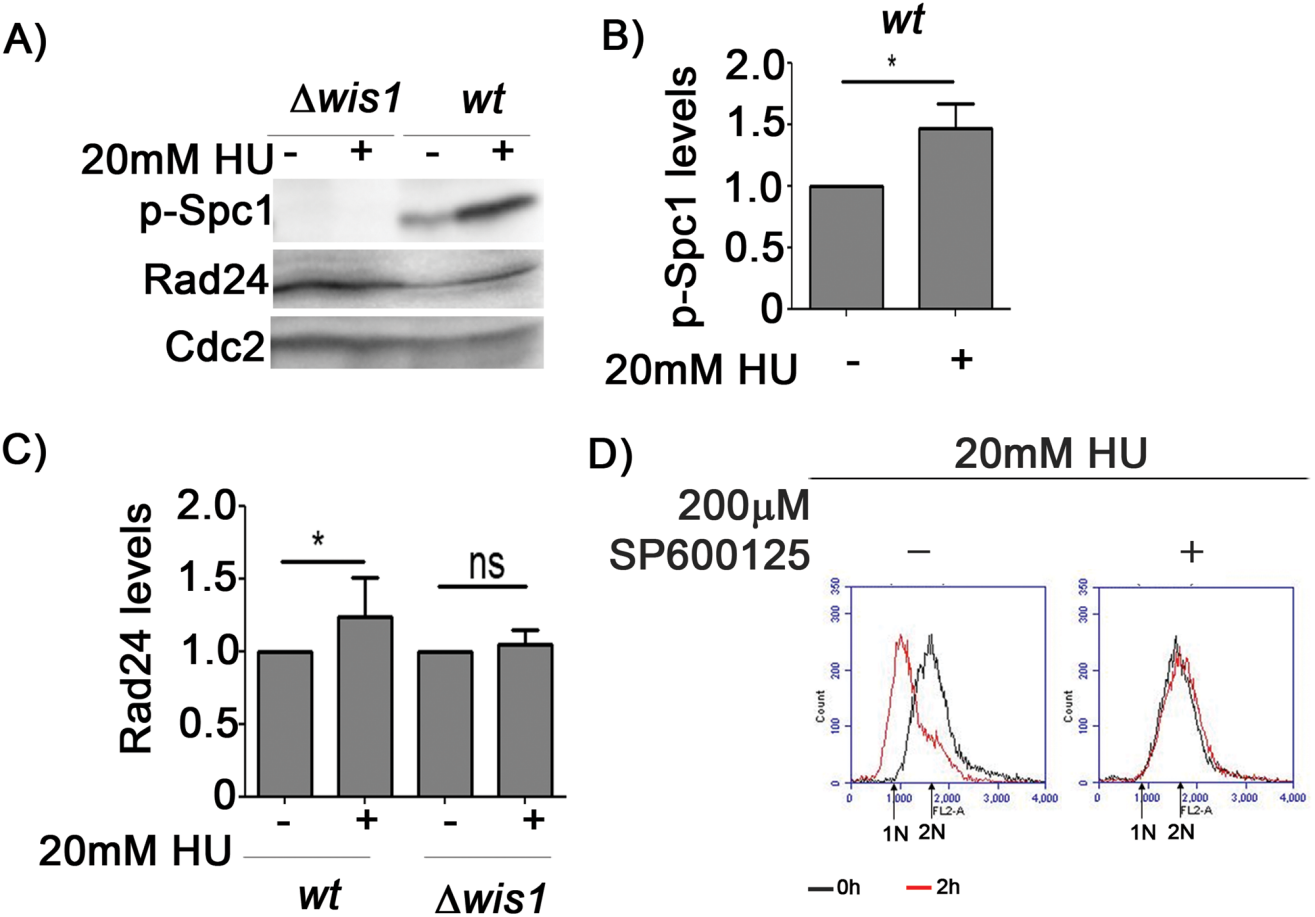
**Spc1 activity is essential for checkpoint activation during nucleotide depletion.** (A) Phospho-Spc1 and Rad24 protein levels in wt and Δ*wis1* after treatment with 20 mM HU for 15 min determined by immunoblotting and (B,C) quantified using ImageJ; *, *P*<0.05. Cdc2 levels were used for normalisation. (D) Flow cytometric analysis of DNA content of wt cells treated with 20 mM HU for the indicated time periods in presence or absence of 200 µM of SP600125 (for Spc1 inhibition). All data are representative of three independent experiments. All bar graphs represent mean±s.e.m. A one-tailed *t*-test was performed for evaluation of statistical significance.

Aberrant Cdc2 activity is known to cause DNA damage in *S. pombe* cells (Caspari et al., 2002; Hills and Diffley, 2014; Deng et al., 2019) and this DNA damage may be the reason for the observed changes in Spc1 activity in mutants with aberrantly high Cdc2 activity. It has been reported that the presence of a hyperactive Cdc2 in *Δwee1* cells causes dNTP depletion and replication stress (Pai et al., 2019). In combination with our results, this pointed to the possibility that the observed Spc1 activation in our experiments might be a response to replication stress. To confirm that possibility, we treated wt *S. pombe* cells with HU (a ribonucleotide reductase inhibitor, known to cause dNTP depletion in *S. pombe*). We found that HU treatment for 15 min does cause an increase in phosphorylated Spc1 levels in wt cells, thus implicating replication stress resulting from nucleotide depletion as another trigger for Spc1 activity (Fig. 6A,B). This activation of Spc1 upon HU treatment also occurs via Wis1 MAPKK, as we found no increase in Spc1 phosphorylation in *Δwis1* mutants (Spc1 levels under these conditions remained unchanged, see Fig. S1E). We checked Rad24 levels under these conditions. In wt cells HU treatment increased Rad24 levels while in *Δwis1* cells no such change was seen (Fig. 6C). HU treatment is known to cause G1/S checkpoint activation in *S. pombe* (Boddy et al., 1998; Walworth et al., 1993; Tormos-Pérez et al., 2016), so we treated the cells with HU for 2 h, which leads to accumulation of cells at the G1-S boundary. We found that pretreating the cells with the Spc1 inhibitor abrogates the activation of this checkpoint (Fig. 6D), which clearly establishes that Spc1 activity is important for G1/S checkpoint activation after nucleotide depletion.

**Fig. 7.**
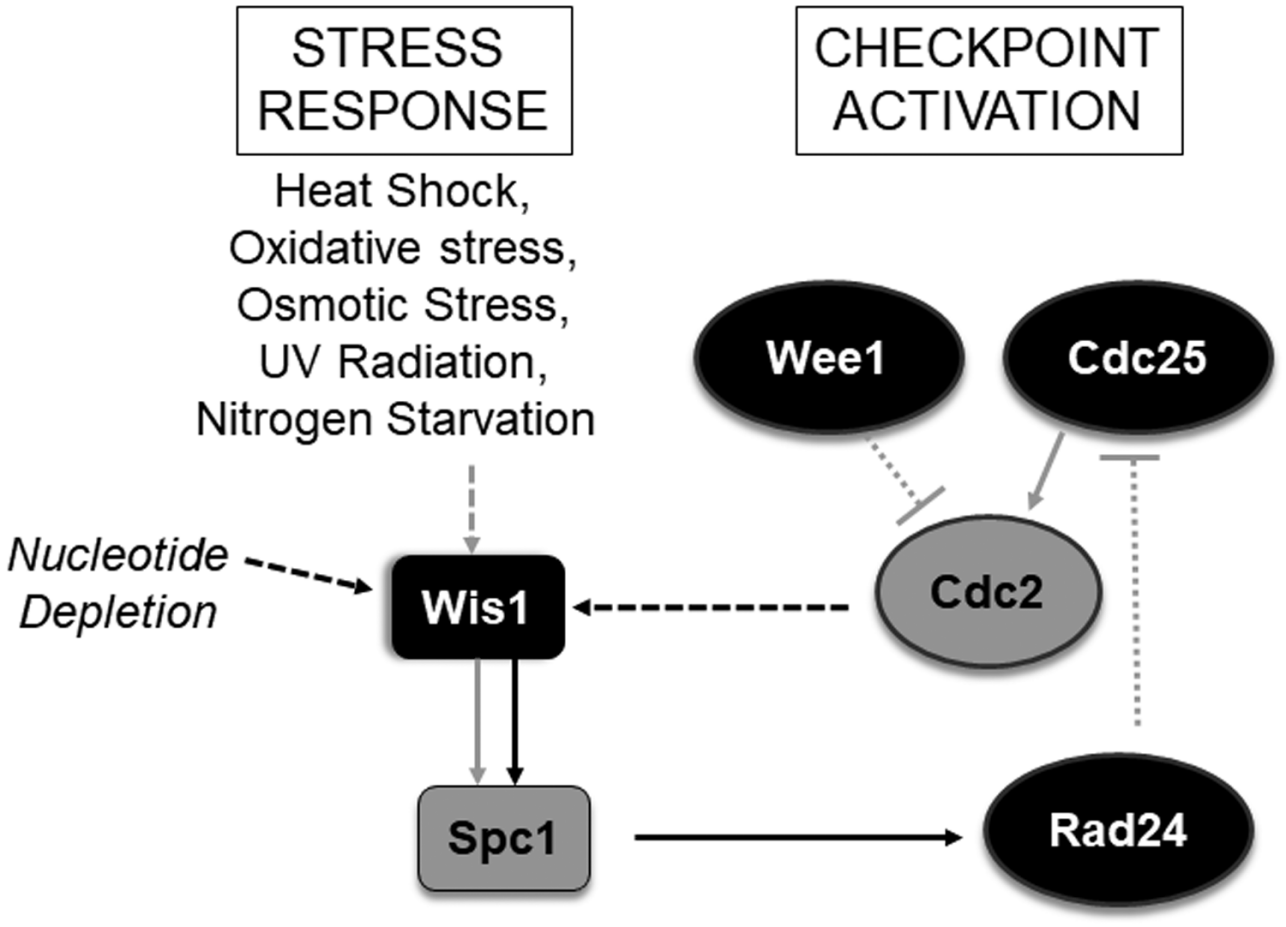
**Model for the association between Cdc2 activity and the Wis1-Spc1 MAPK pathway.** Grey lines represent known connections while black lines represent new connections reported in this work.

Taken together, our results provide evidence for the existence of a pathway through which changes in Spc1 activity in response to changes in Cdc2 activity can control mitotic timing and contribute towards preserving the mitotic fidelity of *S. pombe* cells. (Fig. 7). We show that changes in Cdc2 activity can be communicated to the MAPK Spc1 via the MAPKK Wis1. Hyperactivation of Cdc2 results in enhanced Spc1 activation, while inhibition of Cdc2 results in decreased Spc1 phosphorylation. The increase in Spc1 phosphorylation is much less than that seen during stress conditions, but it is significant enough to cause a change in Rad24 levels and can to some extent protect the cells from mitotic aberrations resulting from Cdc2 hyperactivity. The fact that a decrease in Cdc2 activity can cause decreased phosphorylation of Spc1, both in the presence and absence of stress stimuli, and that the decrease is reversible in nature indicates that robust communication between these two kinases exists, which may either be direct or indirect. We also show that replicative stress arising from HU-inflicted nucleotide depletion can also activate the Wis1-Spc1 MAPK pathway. Spc1 has been shown to be important for *hsp16^+^* expression in cells treated with HU (Taricani et al., 2001), but increase in Spc1 activity in such conditions as well as the requirement of Spc1 for G1/S checkpoint activation has not yet been demonstrated. These new findings add to the importance of Spc1 in the stress response mechanisms in *S. pombe*.

## MATERIALS AND METHODS

### Strains, media and growth conditions

The *S. pombe* strains used in this study are listed in Table 1. Cells were grown as described by Moreno et al. 1991. All cells were grown at 30°C and 25°C (for temperature-sensitive mutants) in yeast extract with supplements (YES) medium unless indicated otherwise. For overexpression experiments, cells were grown overnight in Edinburgh minimal medium (EMM) without leucine, supplemented with 20 μM thiamine, harvested, washed, resuspended in EMM without leucine and incubated for another 24 h (2 h in fresh media after normalizing O.D to 0.8) at 30°C. SP600125 (Sigma-Aldrich) at a concentration of 200 μM was used for inhibition of Spc1 (Hartmuth and Petersen, 2009).

**Table 1.**
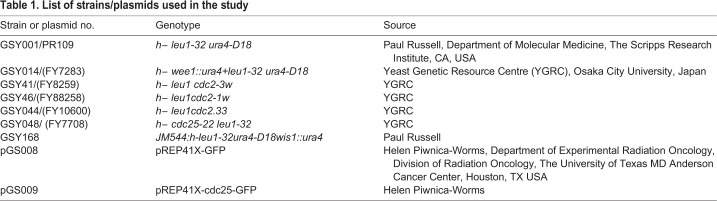
**List of strains/plasmids used in the study**

### Microscopy

*S. pombe* cells were grown as indicated in the standard manufacturers protocol, and examined using an OlympusBX51 Fluorescence Microscope at 100x magnification. Brightfield images of live cells were taken. Cell length analysis was done by measuring the length of septated cells using ImageJ software (Schneider, Rasband, and Eliceiri, 2012). For studying mitotic defects following Spc1 inhibition, cells were stained with DAPI (2 µg/ml final concentration) and examined using an OlympusBX51 Fluorescence Microscope at 100x magnification. All images were taken and processed with the use of identical parameters.

### Statistical analysis

Statistical analysis of all quantitative data was performed using the GraphPad Prism software (www.graphpad.com/guides/prism/7/user-guide/index.htm?citing_graphpad_prism.htm). All bar graphs represent mean±s.e.m. A one-tailed *t*-test was performed for evaluation of statistical significance of all quantitative data and the *P*-values were used to estimate significance of the results: ***, *P*-value <0.001; **, *P*-value <0.01; *, *P*-value <0.05.

### *S. pombe* transformations

A 1mL aliquot of an overnight *S. pombe* culture in YES was harvested and then resuspended in 0.5 ml of 10 mM Tris pH 8, 1 mM EDTA, 0.1 M lithium acetate, 40% PEG (PEGLET). A 5 μl aliquot of denatured salmon sperm DNA (10 mg/ml) was added to it. 1 µg of the purified plasmid DNA was then added to this mixture and allowed to stand overnight at room temperature after which the cells were harvested, resuspended in 150 µl YES and spread on appropriate selection plates.

### Protein extraction and immunoblotting

Whole cell extracts were prepared as described before (Paul et al., 2018). Briefly, cells were harvested and resuspended in 20% Trichloroacetic acid (TCA) and vortexed at maximum speed for five 1 min pulses after adding glass beads. The solution was then transferred to a fresh microcentrifuge tube (to remove the glass beads) and then centrifuged at 13,000 rpm for 15 min. The pellets were then washed with 70% ethanol. All steps were performed at 4°C and samples were kept on ice. The pellets were finally resuspended in Laemmli buffer and boiled for 5 min before being loaded onto 10% SDS–polyacrylamide gels. After transferring onto PVDF membranes, immunoblotting was performed using anti-Cdc2 (Sc-53217) antibody, anti-14–3–3ε (Sc-23957), anti-Phospho p38 (Cell Signalling Technology # 9211S, for Phospho-Spc1) or anti-Spc1 (lab stock) antibodies at 1:1000 dilutions for all and at 1:2500 dilution for the latter. Immunoblots were developed using Advansta WesternBright™ ECL reagent (K-12045-D50).

### Protein level quantification

Quantification of the signals from immunoblot was performed using ImageJ. p-Spc1, Cdc2 and Rad24 immunoblotting was performed on the same blot and hence Cdc2 signals were used for normalisation, except in case of samples from *cdc2-33 ts* mutants. For these mutants, normalisation of Rad24 or p-Spc1 levels was performed using the signal intensity from a non-specific band from the image of the Ponceau stained blot. To determine Spc1 levels, the samples were rerun and Cdc2 immunoblotting was performed on both blots to ensure equal loading of samples.

### RNA isolation and qPCR

Total cellular yeast RNA was isolated from the samples as described in an earlier paper (Paul et al., 2018). About 2 μg of the isolated RNA, after DNaseI treatment, was converted to cDNA using reverse transcriptase [Moloney murine leukemia virus (M-MuLV) reverse transcriptase; Thermo Fisher Scientific]. qPCR was performed in an Applied Biosytems Real Time Fast 7500 instrument using SYBR Green reagent (Applied Biosystems). Melt curve analysis was done to confirm the absence of primer dimers and non-specific amplification products. Primers used for real-time PCR are Rad24: forward: 5′AGTTTGCCGTTGGTGAGAA3′, reverse: 5′AAGCGGATAGGATGAGTAGGT3′; 18S rRNA: forward: 5′TGTACTGTGAAACTGCGAATGGCTC3′, reverse: 5′GCAAGGCCATGCGATTCGAA3′.

### Dead cell quantification by flow cytometry

To quantify the extent of cell death, cells grown under indicated conditions were stained with Propidium Iodide (2 µg/ml) and proportion of dead cells was ascertained by quantifying the number of cells showing Propidium Iodide uptake by flow cytometry using the BD Accuri™ C6 Plus flow cytometer.

### DNA content analysis

For DNA content analysis, cells growing in the indicated conditions were fixed in 70% ethanol. The fixed cells were then washed and rehydrated in 50 mM sodium citrate buffer. The cells were resuspended in 500 μl of 50 mM sodium citrate buffer containing 0.1 mg/ml of RNase A and incubated at 37°C for 4 h. Propidium Iodide (2 μg/ml) was used for DNA staining. DNA content was then measured flow cytometrically using the BD Accuri™ C6 Plus flow cytometer.

## Supplementary Material

Supplementary information
