# Supplementary material for: Communication between Cyclin-dependent kinase Cdc2 and the Wis1-Spc1 MAPK pathway determines mitotic timing in Schizosaccharomyces pombe
Source: Biol Open. 2020 Jul 21;9(7):bio053322. doi: 10.1242/bio.053322 (PMC7390630; doi:10.1242/bio.053322)
Supplement: Supplementary information [file biolopen-9-053322-s1.pdf]

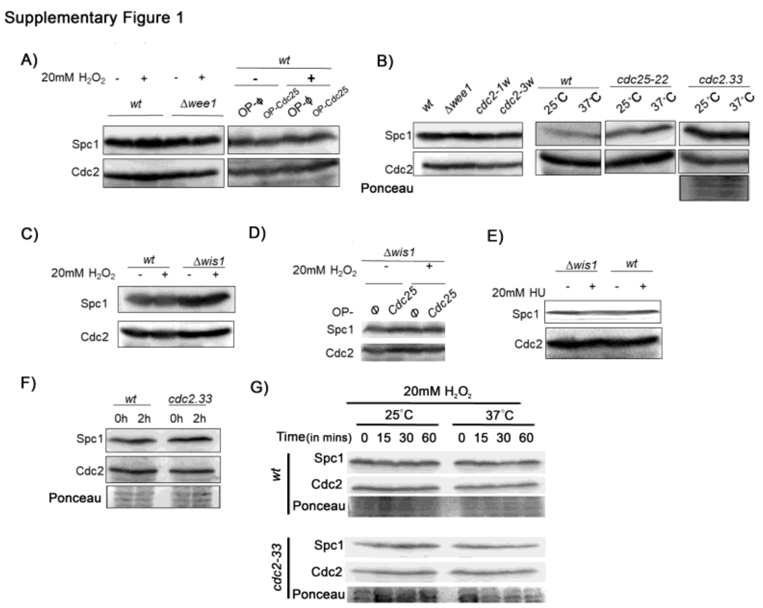

Fig S1. Spc1 levels remain unchanged with variations in Cdc2 activity. (A) Spc1 levels in *wt* and Δ*wee1* cells treated with 20mM H_2O_2 for 15 mins and in *wt* cells overexpressing pGS008(φ) or pGS009(Cdc25-GFP) and treated with 20mM H_2O_2 for 20 mins. Total Spc1 and Cdc2 protein levels in all cases determined by immunoblotting. (B) Spc1 levels in *wt*, Δ*wee1*, *cdc2-1w*, *cdc2-3w* mutants and in *wt* and *cdc25-22*, *cdc2-33* at 25°C and 37^0C (non-permissive temperature) (C) Spc1 levels in *wt* and Δ*wis1* cells treated with 20mM H_2O_2 for 15 mins. (D) Spc1 levels in *wt* and Δ*wis1* cells overexpressing pGS008(φ) or pGS009(Cdc25-GFP) and treated with 20mM H_2O_2 for 20mins. (E) Spc1 levels in *wt* and Δ*wis1* treated with 20mM HU for 15mins. (F) Spc1 levels in *cdc2-33 ts* mutant before (0 hr) and after release into permissive temperature (2 hrs) after being blocked at the non-permissive temperature. (G) Spc1 levels in *wt* and *cdc2-33 ts* mutant after treatment with 20mM H_2O_2 for the indicated time points at the indicated temperatures. Spc1 and Cdc2 protein levels in all cases determined by immunoblotting. Cdc2 levels were used as the loading control except for *cdc2-33 ts* for which total protein levels were used instead. All data are representative of three independent experiments.

Biology Open • Supplementary information

Supplementary Figure 2

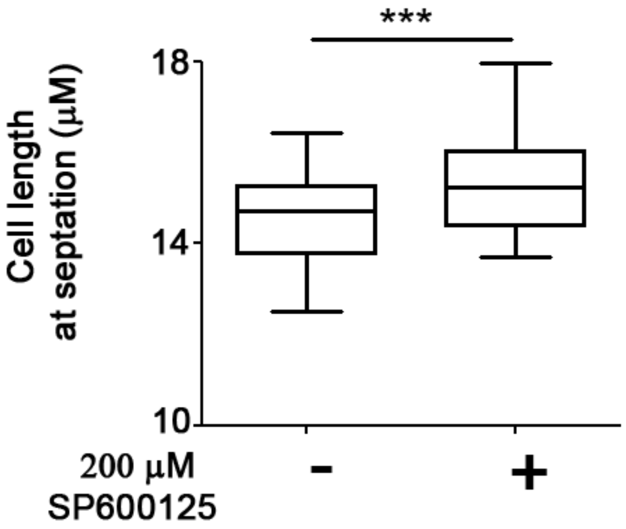

Fig. S2. Quantification of the length at septation of *wt S. pombe* cells before and after treatment with Spc1 inhibitor (SP600125) for 2 hr, using ImageJ software. *** $p < 0.001$ ($n > 100$). All data are representative of three independent experiments.

Biology Open • Supplementary information
